# Bullous Rheumatoid Neutrophilic Dermatosis—A Systematic Review of 28 Cases

**DOI:** 10.3390/jcm15031003

**Published:** 2026-01-26

**Authors:** Ewelina Mazur, Dominika Kwiatkowska, Justyna Szczęch, Dominik Samotij, Adam Reich

**Affiliations:** 1 Department of Dermatology, Faculty of Medicine, Institute of Medical Sciences, University of Rzeszów, al. Tadeusza Rejtana 16C, 35-959 Rzeszow, Polanddominik.samotij@gmail.com (D.S.); 2 Doctoral School, University of Rzeszów, al. Tadeusza Rejtana 16C, 35-959 Rzeszow, Poland

**Keywords:** bullous, rheumatoid neutrophilic dermatosis, rheumatoid neutrophilic dermatitis, RND, vesico-bullous

## Abstract

**Background/Objectives:** Rheumatoid neutrophilic dermatosis (RND) is a rare extra-articular manifestation of rheumatoid arthritis (RA) with variable clinical presentations. Although typically non-blistering, a rare bullous or vesiculobullous subtype has been described, mainly in patients with seropositive and active RA, and may mimic autoimmune blistering diseases. The objective of this review was to systematically summarize the clinical, histopathological, immunopathological, and therapeutic features of vesiculobullous rheumatoid neutrophilic dermatosis. **Methods:** A systematic literature review was conducted in accordance with the PRISMA 2020 guidelines utilizing the PubMed, MEDLINE, and Google Scholar databases, which were searched through December 2025. Case reports and case series describing vesiculobullous or bullous RND with extractable patient-level data were included. Non-English articles were translated. Demographic, clinical, histopathological, immunopathological, microbiological, and therapeutic data were extracted and analyzed using Statistica 12.0 software. **Results:** Results were synthesized descriptively due to clinical heterogeneity and limited sample size. Thirty reported cases were identified, of which 28 non-duplicate cases were included. The mean patient age was 60.8 ± 14.9 years, with a female predominance (male-to-female ratio, 1:2.5). Most patients were of Asian descent (67.9%). Bullous or vesicular lesions most frequently involved the lower legs (64.3%), palms and soles (41.7%), and thighs (35.7%). Rheumatoid factor data were available in 67.9% of patients, all indicating high RA activity. Histopathological examination was reported in 71.4% of cases and most commonly demonstrated a predominantly neutrophilic infiltrate, often dense and extending throughout the dermis, with subepidermal blister formation being the most frequent pattern. Direct immunofluorescence, serological testing for autoimmune bullous diseases, and microbiological investigations were predominantly negative. Dapsone and systemic corticosteroids, alone or combined with RA-specific therapies, were the most commonly used treatments. **Conclusions:** This review represents the most comprehensive synthesis to date focused exclusively on the bullous/vesiculobullous subtype of RND, highlighting key diagnostic features such as neutrophil-predominant histopathology, negative direct immunofluorescence, and favorable response to dapsone.

## 1. Introduction

Rheumatoid arthritis (RA) is a chronic systemic inflammatory disease that may be associated with a wide range of extra-articular manifestations, including cutaneous involvement. Among these, rheumatoid neutrophilic dermatosis (RND) is a rare inflammatory skin condition characterized by sterile neutrophilic infiltration of the dermis [1]. Although uncommon, RND is clinically relevant because it is typically associated with severe, active, and seropositive RA and may serve as a marker of heightened systemic inflammatory activity [2,3]. Classically, RND presents with symmetrical erythematous papules, nodules, plaques, or urticaria-like lesions involving the lower extremities and periarticular regions, particularly the dorsal surfaces of the hands and arms. The disease predominantly affects women, with a reported male-to-female ratio of approximately 1:2, and its prevalence among patients with RA has been estimated at 0.9–1.8% [2,3].

The pathogenesis of RND remains incompletely understood. Current evidence supports an immune-mediated mechanism driven by dysregulated neutrophil recruitment and activation in the context of systemic inflammation [4]. Elevated proinflammatory cytokines and chemokines associated with active RA are thought to promote cutaneous neutrophil accumulation. Histopathologically, RND is characterized by a dense dermal neutrophilic infiltrate in the absence of true vasculitis, although leukocytoclasia may be present. Direct immunofluorescence and serological testing for autoimmune blistering diseases are typically negative, further supporting a non-autoantibody-mediated process [5].

A major diagnostic challenge arises from the considerable overlap between RND and other neutrophilic dermatoses, including Sweet’s syndrome (SS), pyoderma gangrenosum, and erythema elevatum diutinum, as well as infectious conditions and leukocytoclastic vasculitis [4]. Whether RND represents a distinct disease entity or a variant within the spectrum of SS remains controversial. While both disorders share overlapping histopathological features—such as dense neutrophilic infiltrates and dermal microabscess formation—RND is more closely linked to RA disease activity and typically lacks the systemic symptoms, including fever and constitutional manifestations, that are characteristic of SS [5].

Among the recognized clinical variants of RND, the bullous or vesiculobullous subtype is exceptionally rare and poorly characterized. Its clinical resemblance to autoimmune blistering diseases often necessitates extensive immunopathological and microbiological evaluation, contributing to diagnostic delay. Because published data are limited to isolated case reports and small case series, no standardized diagnostic criteria or evidence-based treatment recommendations currently exist.

Accumulating evidence suggests that bullous RND represents a reproducible clinicopathologic phenotype rather than a mere morphological variant, characterized by vesiculobullous morphology, neutrophil-predominant histology, and consistently negative immunopathological studies. Clinically, bullous RND is frequently misdiagnosed as an autoimmune blistering disease, often resulting in delayed diagnosis and unnecessary immunobullous investigations or treatments.

**Figure 1:**
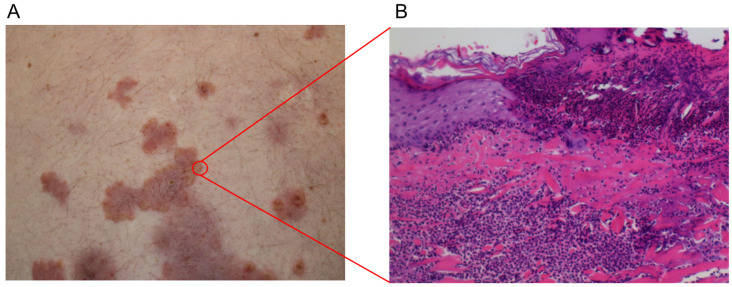
(**A**) Violet papules and plaques on the abdomen showing small vesicles at the periphery of the lesions. (**B**) Erosion and neutrophilic infiltration mainly seen in the dermis, mostly expressed in its upper layers (H&E 100×).

To date, no systematic reviews have focused exclusively on the vesiculobullous subtype of RND. The aim of this study is to systematically review and synthesize the available literature on bullous RND, summarizing the clinical, histopathological, immunopathological, and therapeutic features of 28 published cases. By integrating existing evidence, we highlight that bullous RND represents a distinct but underrecognized manifestation of neutrophilic dermatosis associated with active RA, characterized by neutrophil-predominant histopathology (Figure 1) and consistently negative immunobullous studies, underscoring the importance of considering this entity in the differential diagnosis of blistering eruptions in patients with RA.

## 2. Materials and Methods

**Figure 2:**
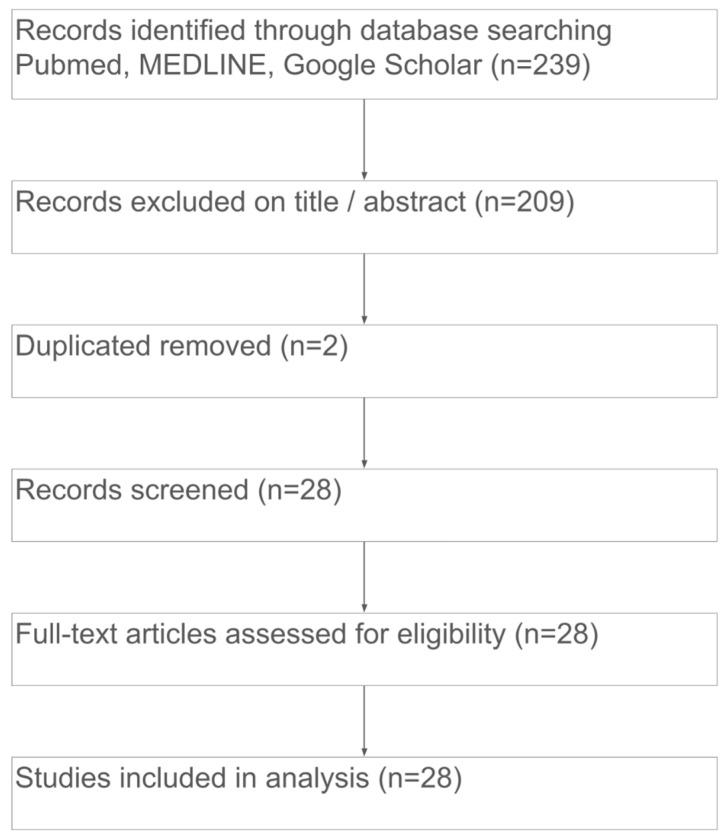
PRISMA 2020 flow diagram illustrating the study selection process for the systematic review of bullous rheumatoid neutrophilic dermatosis.

This review was conducted following the PRISMA 2020 (Preferred Reporting Items for Systematic Reviews and Meta-Analyses) guidelines. Study selection is summarized in a PRISMA 2020 flow diagram (Figure 2). Given the rarity of bullous RND, the methodological aim was to identify and synthesize all available patient-level clinical evidence (case reports and case series) describing vesicular and/or bullous presentations compatible with bullous RND.

### 2.1. Eligibility Criteria

We included publications that met all of the following criteria: (i) human subjects with a clinical diagnosis of rheumatoid neutrophilic dermatosis/dermatitis in the context of rheumatoid arthritis, (ii) vesicles and/or bullae described clinically (including “vesicobullous” or “bullous” variants), and (iii) sufficient patient-level information to extract at least clinical phenotype and treatment/outcome (with histopathology and DIF recorded when available); no restrictions were placed on study design or follow-up duration. We excluded: (i) non-bullous RND without vesicles/bullae, or cases where treatment outcomes were not reported; (ii) review articles without original patient data (these were used only for citation-chaining); (iii) duplicate reports of the same patient (the most complete report was retained); and (iv) abstracts or conference items lacking extractable clinical details.

### 2.2. Information Sources and Search Strategy

We conducted a literature search in December 2025 using PubMed, MEDLINE, and Google Scholar databases for original articles, systematic reviews, case series, and reports related to bullous RND. No language restrictions were applied to the search strategy. The core search terms were: “rheumatoid neutrophilic dermatosis” OR “rheumatoid neutrophilic dermatitis” combined with at least one blistering-related term: “bulla*”, “vesic*”, “blister*”, or “vesicobullous”.

Where supported (e.g., PubMed), terms were searched in Title/Abstract fields and combined with Boolean operators. Reference lists of all eligible full texts and relevant narrative/systematic reviews were manually screened (“backward citation searching”) to capture additional cases not retrieved in the primary search. Forward citation searching (via database-linked “cited by” features when available) was also used to identify later reports referencing seminal bullous RND cases.

### 2.3. Study Selection

Search terms employed were “rheumatoid neutrophilic dermatosis” and “rheumatoid neutrophilic dermatitis” combined with the additional search terms “bulla”, “vesicle”, or “vesico-bullous”. No automation tools were used during study selection. All articles were reviewed to identify additional relevant publications not included in the initial database search. Articles were selected and screened primarily by two authors (E.M. and D.K.). Authors D.S., J.S. and A.R. resolved article selection disputes. A total of 28 articles were selected for this review.

### 2.4. Data Extraction and Operational Definitions

Data were extracted into a standardized spreadsheet designed a priori. Extracted variables included: demographics (age, sex, ethnicity/geographic origin when available), rheumatoid arthritis features (serostatus, rheumatoid factor level if provided, flare/exacerbation descriptors), clinical morphology (vesicles vs. bullae, distribution, mucosal involvement), systemic symptoms, histopathology descriptors (depth and density of neutrophilic infiltrate; intraepidermal vs. subepidermal blister; leukocytoclasia; presence/absence of vasculitis; microabscesses), direct immunofluorescence (DIF) results, serology relevant to autoimmune blistering diseases (when reported), microbiologic studies (culture/PCR), treatment regimens (topical/systemic corticosteroids, dapsone, DMARDs, biologics, combination therapy), and outcomes (resolution/recurrence/scarring, response time when stated).

For synthesis purposes, “bullous RND” was defined as RND in which the clinical description explicitly reported bullae and/or vesicles, regardless of whether the original authors used the terms “bullous,” “vesicobullous,” or “blistering.” Diagnostic certainty was assessed based on clinicopathologic correlation, association with RA, and exclusion of infectious or immunobullous diseases as reported in each case. Potential misclassification between pustular and vesiculobullous lesions was considered during data extraction—skin lesions described by the authors as pustular or visible as pustular in the attached clinical photographs were not included in the analysis. When lesion location was described in multiple anatomic terms, sites were harmonized into pre-specified categories (e.g., lower legs, palms, soles, trunk, upper extremities).

### 2.5. Translation of Non-English Literature

Non-English articles identified as eligible were translated using professional medical translation tools prior to extraction to reduce language bias and to enable consistent variable capture.

### 2.6. Quality Considerations

Given that the evidence base consists predominantly of case reports/case series, formal risk-of-bias scoring was not used to exclude studies. Instead, we focused on completeness of reporting for key diagnostic domains (clinicopathologic correlation, DIF, exclusion of infection/autoimmune blistering disease where appropriate). Missing data were recorded as “not reported,” and denominators were adjusted accordingly for each variable.

### 2.7. Statistical Analysis

Data were analyzed using Statistica 12.0 Software for Windows Software. Continuous variables were summarized as mean ± standard deviation (or median and range when appropriate, based on reporting). Categorical variables were summarized as counts and percentages. Because of heterogeneity in reporting and the small number of cases, absence of control groups, analyses were descriptive; no meta-analytic pooling or hypothesis testing was planned. Formal risk-of-bias tools were not applied, as all included studies were case reports or small case series; instead, completeness of diagnostic reporting was assessed.

## 3. Results

The literature review identified 30 reported cases of bullous RND: 17 from the English-language literature (with two duplicates) and 13 from foreign literature (seven Japanese, four Chinese, one Spanish, and one French). Non-English articles were translated. The final review included 28 studies (Figure 2). The complete list of publications and patients’ data included in this review are summarized in Table 1.

### 3.1. Study Population

The patients’ mean (±standard deviation) age was 60.8 ± 14.9 years. Females comprised the majority of the population, and the male-to-female ratio was 1:2.5. Most of the patients with bullous RND were Asian (67.9%), followed by Caucasian (21.4%), Hispanic (7.1%), and African (3.6% each) races.

### 3.2. Lesion Localization and Disease Severity

The most common sites of bullous or vesicular lesions were the lower legs (*n* = 64.3%), palms and soles (41.7%), and thighs (35.7%). Rheumatoid factor data were available in 19 of 28 (67.9%) patients.

Rheumatoid factor positivity was interpreted as a surrogate marker of active or severe RA, acknowledging that formal disease activity scores (e.g., DAS28) were not consistently reported. All patients with available data (100%) demonstrated high RA activity (RF > 15 IU/mL or positive), while RF status was not reported in 9 cases (32.1%).

### 3.3. RND Treatment

Among the 28 cases reporting treatment strategies, dapsone was the most frequently used therapy, administered as monotherapy in 9 cases (32.1%), with doses ranging from 75 to 150 mg/day, most commonly 100 mg/day. Combination therapy consisting of systemic corticosteroids plus an immunomodulatory or RA-specific agent (including methotrexate, tretinoin, colchicine, leflunomide, sulfasalazine, abatacept, etanercept, or hydroxychloroquine) was used in 8 cases (28.6%). RA-specific agents without concomitant systemic corticosteroids were employed in 3 cases (10.7%), while systemic corticosteroids alone were used in 2 cases (7.1%). Topical corticosteroids alone were reported in 3 cases (10.7%). Two reports (7.1%) described either spontaneous resolution or unspecified RA-directed therapy.

### 3.4. Histopathology

Twenty (71.4%) out of 28 articles depicted histopathological features of biopsy specimens obtained from bullous lesions. The most pronounced finding was a predominantly neutrophilic infiltrate, observed in 70.0% of specimens. Dense neutrophilic infiltration was described throughout the dermis in 45.0% of cases and was confined to the upper dermis in 20.0%. Blister formation was reported in 57.1% of cases, most commonly subepidermal (39.3%), followed by intraepidermal blistering (21.4%); one case demonstrated both intraepidermal and subepidermal blistering. Leukocytoclasia in the absence of vasculitis was present in 28.6% of cases, while dermal microabscess formation was reported in 7.1%.

### 3.5. Direct Immunofluorescence

Direct immunofluorescence studies were performed in 12 (42.9%) of the 28 included articles. Among these, the vast majority yielded negative results (91.7%). In one case, slight C3 deposition within blood vessel walls was observed.

### 3.6. Autoantibodies

Serological investigations for autoimmune bullous diseases, including enzyme-linked immunosorbent assays for BP180 antibodies and circulating autoantibodies, were reported in four cases (14.3%); all yielded negative results.

### 3.7. Additional Studies

Microbiological investigations, including tissue cultures, periodic acid–Schiff staining, or microorganism identification, were performed in 7 cases (25.0%) and yielded negative results in all instances. In one case, a lesional swab tested negative for herpes simplex virus DNA by polymerase chain reaction.

### 3.8. Limitations

This study has several limitations. The analysis is based primarily on case reports and small case series, which are inherently subject to publication bias and heterogeneity in diagnostic work-up, clinical description, and follow-up. Important variables, including rheumatoid arthritis activity, serological findings, histopathological features, direct immunofluorescence, microbiological investigations, and treatment outcomes, were not consistently reported across all cases, resulting in variable denominators for pooled analyses. Therapeutic approaches were highly heterogeneous, limiting assessment of treatment efficacy, and long-term outcomes were rarely documented. Finally, as this review is limited to published cases, milder or underdiagnosed presentations are likely underrepresented.

Overall, bullous RND predominantly affected older women with active RA and was characterized by neutrophil-predominant histopathology, negative immunopathological studies, and favorable response to anti-neutrophilic therapies.

The predominance of Asian patients likely reflects publication bias rather than true epidemiologic distribution.

## 4. Discussion

RND is a rare skin disease associated with severe seropositive RA. As of 2024, around 54 cases of this disease have been reported in the literature [21,32]. RND typically manifests as asymptomatic papules, nodules, or plaques predominantly on the extremities and is histologically characterized by a sterile, dense dermal neutrophilic infiltrate without vasculitis [32]. Classic presentations are non-bullous, and according to Scotti et al. [32], vesiculobullous or pustular subtypes constitute a minority (≈12.7%) of reported RND cases and pose particular diagnostic challenges due to their overlap with other neutrophilic dermatoses and autoimmune blistering disorders. However, given that this article presents only a bullous subtype of RND and includes 28 cases of this disease entity, it is possible that the literature data on the prevalence of this condition is underestimated. This is in accordance with the study conducted by Kakurai et al., in which vesiculobullous lesions were found in 25.6% of RND patients [31].

Histologically, neutrophilic dermatoses (NDs) can be divided into epidermal and primary dermal, based on the location of the neutrophilic infiltrate [33,34,35]. Sneddon-Wilkinson disease and IgA pemphigus are epidermal and bullous NDs [36]. Dermatitis herpetiformis (DH), linear IgA bullous dermatosis (LABD), and bullous systemic lupus erythematosus (BSLE) are primary dermal ND with no vasculitis and blistering [33]. SS is also a primary dermal ND, with only a few bullous cases reported in the literature [37]. RND has no obvious clinical manifestations and histopathological specificity, so it is important to differentiate it from other NDs, such as SS, pyoderma gangrenosum, or Behcet’s syndrome [38]. Some scholars believe that RND is a variant of SS with similar histologic manifestations. Still, SS is often accompanied by systemic symptoms such as fever, malaise, arthralgia, conjunctivitis, etc., whereas RND is usually free of systemic symptoms [39]. Bullous SS (BSS) is a rare presentation of this disease entity. It presents as flaccid or tense blisters at classical SS sites, including the extremities, trunk, and face [40]. BSS was reported in the literature as a disease associated with (a) malignancy [41,42], (b) ulcerative colitis [43,44], (c) drugs [45], (d) ANCA-positive vasculitis [45,46], or (e) a lack of underlying disease [47]. As mentioned before, BSLE, LABD, and DH are the main differential diagnoses for RND because they all display primary dermal ND with blister formation but no vasculitis in histology.

BSLE was defined in 1995 by Yell et al. as “an acquired subepidermal blistering disease in a patient with SLE, with immune reactants at the basement membrane zone (BMZ) on either DIF or indirect immunofluorescence” [48]. Later, de Risi-Pugliese et al. [49] collected 128 cases of BSLE, which exhibited tense bullae on normal or erythematous skin, primarily localized on the trunk, arms, head, and neck. On histology, sub-epidermal detachment, dermal infiltration of polynuclear neutrophils aligned at the BMZ, and leukocytoclasia were seen. DIF displayed a variety of patterns exhibiting linear and/or granular deposits of IgG, IgA, IgM, and/or C3 at the BMZ. Anti-type VII collagen antibodies were found in 69% of cases of BSLE. Dapsone proved effective in 90% of instances [49].

Dermatitis herpetiformis (DH) is an intensely pruritic bullous dermatosis and one manifestation of coeliac disease, with epidermal transglutaminase (TG3) as an autoantibody [50]. A symmetric distribution characterizes DH, and the lesions affect the extensor surfaces, including the elbows, dorsal forearms, knees, and buttocks [51]. Pathognomonic granular immunoglobulin A (IgA) response in the dermis must be present and directed against TG3 for DH diagnosis. There is also the genetic predisposition, specifically HLA DQ2 or DQ8 haplotypes [51]. The histopathological examination of skin lesions reveals the presence of neutrophils infiltrating the skin, causing the breakdown of proteins in the BMZ. This, combined with the impaired function of type IV collagen and laminin, leads to damage to the anchor fibers and the formation of blisters [52]. The primary approach to treating DH involves strict adherence to a gluten-free diet (GFD) for the duration of the individual’s life. Typically, when GFD alone is incorporated, it may take several months to several years to achieve remission [50]. Consequently, during the initial 1–2 years following diagnosis or during periods of disease exacerbation, medications such as dapsone (25–400 mg/day), other sulfonamides (sulfasalazine/sulfapyridine 1–2 g/day), or topical corticosteroids (betamethasone valerate, betamethasone dipropionate, or clobetasol propionate) can serve as beneficial short-term additional treatment option until the diet alone proves to be sufficient [53].

LABD is a rare (0.5 new cases per million in Western Europe) autoimmune blistering disorder that may be idiopathic, drug-induced, malignancy-associated, or inflammatory bowel disease-associated [54]. LABD presents two age-related peaks of incidence: during early childhood and in individuals aged 60 and above [55]. In the pediatric population, this condition is known as chronic bullous disease of childhood. Women have a slightly higher prevalence of this condition, although its occurrence is fairly similar between genders [55]. The clinical presentation of LABD typically includes tense arciform blisters arranged in a “string of pearls” configuration, often arising on an erythematous/urticarial base. In children, LABD commonly affects the anogenital area and lower abdomen, while in adults, the trunk and extensor surface of the limbs are more commonly involved [56]. Histologically, LABD presents subepidermal blisters associated with dermal infiltration of neutrophils. Linear deposition of IgA along the BMZ in DIF is a defining characteristic of LABD [57]. Individuals with LABD may also have circulating IgA anti-BMZ antibodies [57]. Dapsone 50–150 mg/day is the first-line treatment for adult patients with LABD (0.5–2 mg/kg body weight/day for children). In glucose-6-phosphate dehydrogenase-deficient patients, oral corticosteroids alone (0.5–1 mg/kg body weight/day in the pediatric population, 1 mg/kg body weight/day in adults) may be utilized, even in drug-induced cases [58].

Given the information mentioned above, it is possible to distinguish RND from BSLE, DH, and LABD through DIF examination, as RND exhibits negative findings in DIF.

The case–literature synthesis by Kakurai et al. provides one of the largest comparative analyses of bullous versus non-bullous RND, proposing five diagnostic criteria for RND that bridge clinical and histopathologic features: definitive RA diagnosis, high disease activity, presence of erythematous papules or bullae, predominance of neutrophils without leukocytoclastic vasculitis on biopsy, and microbial sterility [31]. Their findings suggest that bullous RND is more common among patients with prolonged RA duration and seropositive status compared with non-bullous forms, and that tense vesiculobullous lesions on the extremities are a defining clinical characteristic. This work importantly distinguishes bullous RND from clinicopathological mimics such as Sweet’s syndrome, pyoderma gangrenosum, erythema elevatum diutinum, and rheumatoid vasculitis on the basis of subtle but critical features in presentation and histology [31]. Our findings largely support the diagnostic criteria proposed by Kakurai et al. [31], while further emphasizing the consistency in describing neutrophil-predominant histopathology and negative immunopathological studies, while further highlighting the reproducibility of these features in vesiculobullous cases.

When looking into the pathogenesis of cutaneous manifestations in neutrophil-mediated dermatoses, Marzano et al. [59] studied immunohistochemical markers associated with inflammation, including CD3, CD163, tumor necrosis factor-alpha, interleukin 8, interleukin 17, matrix metalloproteinase-9, and myeloperoxidase. They found that those proinflammatory proteins were notably elevated in patients with SS, particularly those suffering from the bullous variant. This overexpression corresponded to extensive neutrophil activation, chemotaxis, and increased tissue degradation, as opposed to the papulonodular pattern of Sweet’s syndrome [59].

Despite these advances, the pathophysiology of RND remains poorly understood. Proposed pathogenic mechanisms are inferred from related neutrophilic dermatoses, as direct mechanistic studies in bullous RND are currently lacking. Evidence points to neutrophil chemoattractants (e.g., IL-8 and other chemokines) derived from inflamed synovial tissue contributing to cutaneous recruitment and activation, potentially explaining the link between high RA activity and skin involvement [29,32]. The presence of tense bullae without significant surrounding erythema and in the absence of immune deposits or vasculitis further supports a neutrophilic, rather than immune complex-mediated, mechanism in bullous RND [31]. Clinically, recognition of this phenotype is crucial, as management may differ—systemic corticosteroids, dapsone, and biologic DMARDs have shown benefit in individual reports, with some evidence suggesting parallel improvement in joint and skin disease activity when systemic anti-inflammatory therapy is optimized [32].

Collectively, these findings support vesiculobullous rheumatoid neutrophilic dermatosis as a distinct neutrophilic dermatosis associated with rheumatoid arthritis. This entity should be included in the differential diagnosis of blistering eruptions in patients with RA, particularly in the context of seropositive disease and high systemic inflammatory activity. Future studies are needed to refine diagnostic criteria further, delineate pathogenic mechanisms, and establish evidence-based therapeutic algorithms for this rare but clinically significant presentation.

## 5. Limitations

This review is limited by the exclusive inclusion of case reports and small case series, which increases susceptibility to publication and reporting bias. Heterogeneity in diagnostic criteria, clinical descriptions, and outcome reporting reflects the lack of standardized definitions for vesiculobullous rheumatoid neutrophilic dermatosis and precluded formal comparative analyses. Owing to the descriptive nature of the available data and the absence of controlled studies, formal grading of the certainty of evidence was not performed. In addition, reliance on published literature may underrepresent milder or unrecognized cases, potentially skewing the observed clinical spectrum.

## 6. Conclusions

Bullous RND is a rare skin disease that usually manifests itself with erythematous vesicles or bullae on the lower legs, palms, soles, and thighs. It mostly affects Asian and Caucasian females with exacerbation of seropositive RA. Histologically, dense neutrophilic infiltrate is seen throughout the dermis or in its upper part. Additional studies, such as DIF, circulating autoantibodies, or tissue cultures, are negative. Early recognition of bullous RND may facilitate timely initiation of appropriate anti-inflammatory or RA-directed therapy. Available reports suggest a generally favorable outcome with minimal scarring, although long-term follow-up data remain limited. RND should be considered in the differential diagnosis of patients with blistering disease, especially if they have seropositive RA and do not have tissue-associated or circulating autoantibodies.

## Figures and Tables

**Table 1:** Rheumatoid neutrophilic dermatosis cases presenting with blisters and/or bullae.

Citation	Age	Sex	Localization	RA Activity	Previous RA Treatments	Treatment of RND
Lowe L. et al. [6]	56	F	Elbows, forearm, thigh, palms, soles	1:320	NSAIDS, hydroxychloroquine sulfate, gold, systemic GCS	Spontaneous resolution
Kurose K. et al. [7]	42	F	Lower legs, palms, soles	6231 IU/mL	PSL (5 mg/day), mizoribine (150 mg/day), NSAIDS (diclofenac sodium 100 mg/day)	No data
Lu CI et al. [8]	35	M	Elbow, knee, forearm lower extremity	Positive	Low-dose PSL (10 mg/day), NSAIDS (naproxen), MTX, penicillamine, sulfasalazine, and cyclosporine	Dapsone 100 mg/day
Kreuter A. et al. [4]	78	F	Lower legs	67.5 IU/mL	MTX low dose	PSL 30 mg/day plus Etanercept 2 × 25 mg/week
Ueda L. et al. [9]	76	F	Trunk, extremities	No data	Systemic GCS, MTX	Dapsone 150 mg/day
Takahashi H. et al. [10]	76	F	Lower legs	631 IU/mL	Sulfasalazine, sodium aurothiomalate	Sulfasalazine
Yamamoto T. et al. [11]	65	F	Lower legs	No data	Low-dose PSL and NSAIDS	Topical GCS
Ishikawa A. et al. [12]	64	F	Lower legs, arm	250 IU/mL	PSL (7 mg/day), MTX (6 mg/week), cyclosporin (100 mg/day)	Topical GCS
Yiping M. et al. [13]	62	F	Generalized	Positive	Not specified	MPSL 8 mg 3 times/day, Tretinoin 20 mg 2 times/day.
Yali S. et al. [14]	59	M	Palms, soles	687 IU/L	No data	MPSL 50 mg/day, Azithromycin 500 mg/day for 1 week, Tripterygium wilfordii 60 mg/day and colchicine 1 mg/day
Fujio Y. et al. [1]	78	F	Lower legs, arms, back	87 IU/mL	PSL (5 mg/day), etanercept (25 mg/2 weeks)	Dapsone 75 mg/day
Soza GM. et al. [15]	56	F	Thighs, soles, feet	No data	MTX, etanercept, adalimumab, rituximab, tocilizumab, certolizumab pegol	Dapsone 100 mg/day
Shin JM. et al. [16]	64	F	Lower legs, palms, soles	158 IU/mL	MTX (10 mg/week), systemic GCS (5 mg/day)	Dapsone 100 mg/day
Kubota N. et al. [17]	68	F	Elbows, soles	428 IU/mL	Systemic GCS, tocilizumab	Topical steroid (not reported)
Masuda K. et al. [18]	77	M	Lower legs, soles, hands	No data	Systemic GCS, bucillamine	Dapsone 75 mg/day
Kosumi H. et al. [19]	78	F	Lower legs	No data	PSL (10 mg/day), salazosulfapyridine, abatacept	PSL (20 mg/day), salazosulfapyridine, abatacept
Gao Y-l. [20]	60	M	Thighs, lower legs	728 IU/mL	Not specified	MPSL i.v. 40 mg/day, oral tretinoin tablets 2 × 20 mg/day, hydroxychloroquine sulfate tablets 2 × 200 mg/day
Kumari I. et al. [21]	21	F	Lower legs, thighs, buttocks, soles, forearms	1:340	No previous medication	PSL 20 mg/day, MTX 10 mg/week, topical GCS—clobetasol propionate
Yang Z. et al. [22]	31	F	Generalized	60.7 IU/mL	Systemic GCS	MPSL 40 mg i.v., changed to prednisone acetate 30 mg orally after 6 days
Chaabouni R. et al. [23]	60	F	Lower legs, ankles	140 IU/mL	Systemic GCS, MTX	Dapsone 100 mg/day
Navarro-Triviño FJ. et al. [24]	79	F	Forearms, dorsal aspect of the hands	No data	MTX, azathioprine, systemic GCS	PSL 1 mg/kg plus leflunomide 100 mg/day for three days, later 20 mg/day
Ha DL. et al. [25]	45	F	Knee, dorsum of the hands, soles	No data	NSAIDS, MTX	RA treatment—unspecified
Wang Z. et al. [26]	60	M	Palms, arms, elbow, axillae, lower legs, buttocks	421 IU/mL	Systemic GCS and other unspecified immunosuppressive agent	Dapsone 100 mg/day
Manriquez J. et al. [27]	54	F	Back	Positive	No data	60mg/d of prednisone
Jost M. et al. [28]	60	F	Arms, hands	No data	MTX, cyclosporine, tocilizumab, rituximab, systemic GCS, sarilumab	Tofacitinib
Mazur E. et al. [29]	61	M	Trunk, upper extremities, thighs	Positive	Systemic GCS, Leflunomide	Pulse of i.v. MPSL (two infusions of 500 mg each), followed by oral MPSL at 4 mg/day and subcutaneous MTX at a dose of 15 mg/week
Sivaprakasam T. et al. [30]	67	M	Lower legs, buttocks, genital area, lower abdomen, bilateral elbows, forearms, and hands	No data	MTX, leflunomide, hydroxychloroquine, golimumab, etanercept, tocilizumab	Etanercept 50 mg/week
Kakurai M. et al. [31]	70	F	Lower legs, buttocks, soles, genital areas	1712 IU/mL	PSL 5 mg/day, tacrolimus hydrate 2 mg/day	Dapsone 75 mg/day

F—female, GCS—glucocorticoids, i.v.—intravenous, M—male, MPSL—methylprednisolone, MTX—methotrexate, NSAIDS—non-steroidal anti-inflammatory drugs, PSL—prednisolone, RA—rheumatoid arthritis, RND—Rheumatoid neutrophilic dermatosis.

## Data Availability

The datasets generated during and/or analyzed during the current study are available from the corresponding author upon reasonable request.
